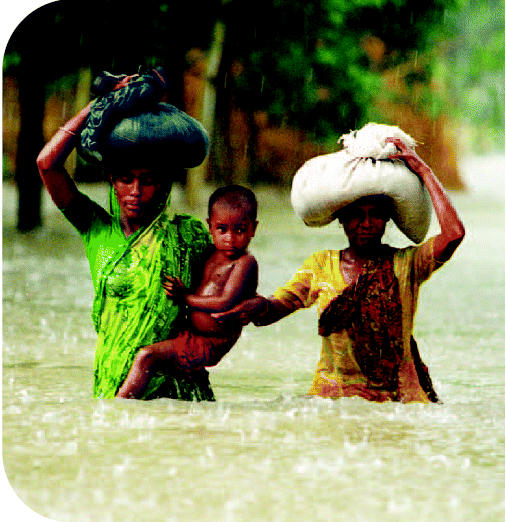# The Beat

**Published:** 2004-09

**Authors:** Erin E. Dooley

## Scratching Out Data on Animal Antibiotic Effects

Farmers use antibiotics to help keep livestock healthy and make them grow faster. Because of concerns that this practice encourages microbial resistance to these drugs, the GAO studied research needs and federal agency efforts on the problem. The GAO’s April 2004 report found that agencies lack the data on linkages between antibiotic use in animals and emerging resistant bacteria that are needed to support research on human health risks. It recommended that the FDA expedite risk assessment of drugs used in animals that are also critical for human health, and that a plan be developed and implemented to fill data gaps in this area.

## Counting Hydrocarbs to Curb U.S. Oil Hunger

In analyzing U.S. fossil fuel consumption, a Cornell University team has determined that energy conservation, along with the development and implementation of energy-efficient technologies, could save consumers $438 billion per year by 2014; conserve chemicals, paper, lumber, and metals; and reduce energy consumption by 33%—just over the amount provided by annual U.S. oil imports. In the June 2004 issue of *Environment, Development, and Sustainability*, the team reported that government subsidies of traditional energy industries, which cost American families about $410 each year in taxes, keep fuel prices artificially low, thus encouraging greater consumption and importation.

## Obesity Report Cards

In June 2004, as part of a state antiobesity program, the nonpartisan Arkansas Center for Health Improvement began mailing annual health reports to the parents of all 450,000 Arkansan public school students. Schools submit each child’s weight and body mass index to the center, which then notifies parents of their child’s weight category and provides healthy lifestyle tips. The center found that 40% of the state’s children are either overweight or at risk for becoming so. Arkansas has also banned vending machines from elementary schools and set up school nutrition, exercise, and child health advisory committees.

## Protein Discovery Sparks Hope for Malaria Vaccine

An international team of researchers reports finding a protein, PfEMP1, on the surface of red blood cells in young children infected with severe malaria, a major cause of morbidity and mortality among children in sub-Saharan Africa. This variant surface antigen could be the target for a vaccine to help children build up antibodies against the disease.

PfEMP1 is not found in other forms of malaria or in older people. Like other variant surface antigens, it enables infected cells to remain in the blood stream and reproduce, rather than being removed by the spleen. The report was published 3 May 2004 in *The Journal of Experimental Medicine.*

## Renewed Commitment to Renewables

At June’s Renewables 2004 conference, a follow-up to the 2002 World Summit on Sustainable Development, representatives from 154 governments pledged anew to promote alternative energy sources, and the World Bank announced it will double loans for renewables projects by 2010. A total of 192 commitments were announced. Currently renewables make up only 5% of the world’s energy supplies.

Meeting attendants also adopted a political declaration, including a vision for equitable access to energy and increased energy efficiency. UNEP estimates that some 1.6 billion people do not have access to electricity. UNEP director Klaus Töpfer cited “energy poverty” as contributing to poverty overall and the associated environmental degradation.

## Floods: Double the Devastation

Today, 25,000 people worldwide are killed each year by flooding, and many more face homelessness, disease, and crop failure following such catastrophes. Blaming such factors as deforestation, climate change, and population growth, United Nations University researchers announced in June 2004 that the number of people affected by devastating floods will double to 2 billion by 2050. Weather-related disasters cost the global economy $50–60 billion annually, and developing countries face the highest relative death toll from these disasters.

## Figures and Tables

**Figure:**
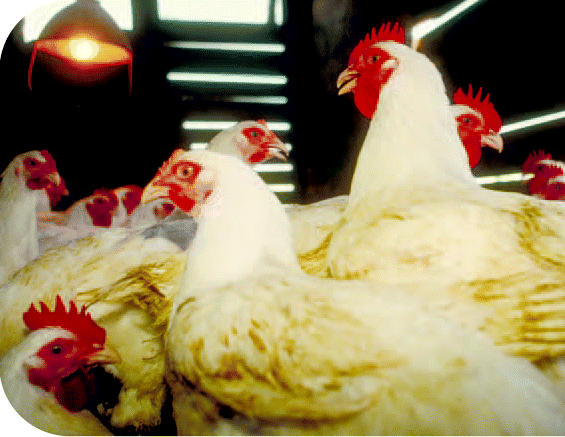


**Figure:**
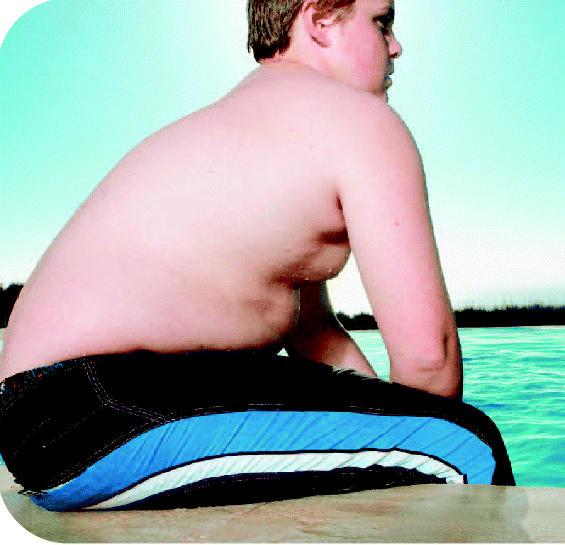


**Figure:**
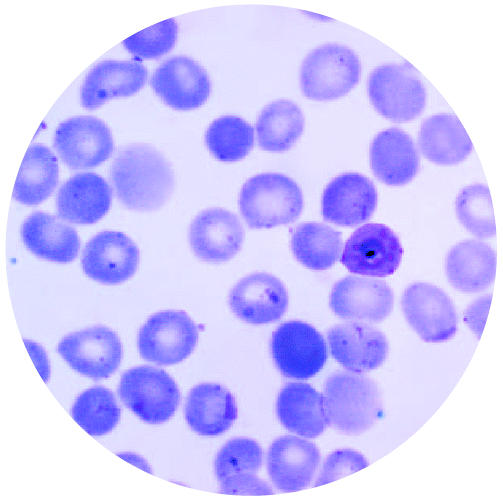


**Figure:**